# Effects of Feeding High-Moisture Corn on Meat Performance, Meat Quality, Muscle Metabolism, and Gut Microbiota in Kazakh Rams

**DOI:** 10.3390/ani16091387

**Published:** 2026-05-01

**Authors:** Buweiaizhaer Maimaitimin, Linhai Song, Kadeliya Abudureyimu, Subinuer Abuduli, Tong Li, Yuxin Zhou, Liang Yang, Wei Shao, Zhijun Zhang, Wanping Ren

**Affiliations:** 1 College of Animal Science, Xinjiang Agricultural University, Urumqi 830052, China; 17590513307@163.com (B.M.); 15009360416@163.com (L.S.); 13699915053@163.com (K.A.); 13649970090@163.com (S.A.); 13853415007@163.com (T.L.); zyxgy1123@163.com (Y.Z.); yangliangagu@xjau.edu.cn (L.Y.); dksw@xjau.edu.cn (W.S.); 2 Institute of Feed, Xinjiang Academy of Animal Husbandry Sciences, Urumqi 830011, China; zzj850916@sina.com

**Keywords:** high-moisture corn, shear force, fatty acid profile, metabolomics, 16S rRNA sequencing, Kazakh rams

## Abstract

This study investigated the effects of feeding High-Moisture Corn (HMC) on meat production performance, mutton quality, muscle metabolism, and gut microbiota in Kazakh rams. Thirty-two 6-month-old Kazakh rams were divided into a control group (CT) and an experimental group (GS). Both groups received a basal diet consisting of 30% whole-plant corn silage, 30% cotton residue, and a concentrate mixture. In the CT group, the concentrate contained 24% ordinary crushed corn (on a dry matter basis). In the GS group, half of the ordinary crushed corn was replaced with HMC, resulting in a concentrate containing 12% ordinary crushed corn and 12% HMC. After a 120-day feeding period, backfat thickness was significantly higher in the GS group (*p* < 0.05); For meat quality, muscle shear force was significantly lower (*p* < 0.01) and intramuscular fat content was significantly higher (*p* < 0.01) in the GS group; Amino acid analysis showed that aspartic acid content was significantly lower (*p* < 0.01), arginine and glutamine contents were significantly lower (*p* < 0.05), and glycine content was significantly higher (*p* < 0.05) in the GS group; Fatty acid analysis revealed that the contents of methyl undecanoate, methyl myristate, methyl palmitate, methyl heptadecanoate, methyl alpha-linolenate, and all-cis-4,7,10,13,16-docosapentaenoic acid were significantly higher in the GS group (*p* < 0.01), while the contents of ten other fatty acids, including methyl caprate, methyl laurate, and methyl tridecanoate, were significantly higher (*p* < 0.05); A total of 668 metabolites were detected by muscle metabolomics, and 20 of them were identified as significantly differential metabolites, with the GS group showing 15 upregulated and 5 downregulated, mainly enriched in four pathways: valine, leucine and isoleucine biosynthesis; taurine and hypotaurine metabolism; pantothenate and CoA biosynthesis; and the citrate cycle (TCA cycle); Gut microbiota analysis showed no significant difference in alpha diversity, but beta diversity was significantly separated between the two groups (*p* < 0.01); Correlation analysis revealed that *Firmicutes_A* was significantly negatively correlated with most fatty acids, while *Proteobacteria* was significantly positively correlated with multiple fatty acids (*p* < 0.05). In conclusion, The GS group had significantly increased backfat thickness, reduced muscle shear force, increased intramuscular fat content, and significantly enriched beneficial fatty acids in Kazakh rams, thereby improving meat quality.

## Simple Summary

High-moisture corn (HMC) is a cost-effective feed for livestock, but its effects on sheep raised for meat are not fully understood. This study investigated the impact of replacing half of the ordinary crushed corn in the diet of Kazakh rams with HMC. After a 120-day feeding trial, we found that the HMC diet significantly improved several aspects of meat quality. The meat from rams fed HMC was much more tender, with a 55% reduction in shear force, and contained a 56% increase in intramuscular fat content, which enhances flavor and juiciness. Furthermore, the meat had higher levels of beneficial omega-3 fatty acids. By analyzing muscle tissue and gut bacteria, we linked these improvements to changes in the animals’ energy metabolism and an increase in beneficial gut microbes associated with fat synthesis. These findings suggest that feeding HMC is a practical strategy for farmers to produce higher-quality, more nutritious lamb while potentially lowering feed costs, offering a win-win for producers and consumers alike.

## 1. Introduction

Kazakh sheep are an important indigenous meat-type breed in the Xinjiang region of China. They are dual-purpose for meat and fat production and are characterized by their hardiness, strong adaptability, fine, tender, and juicy meat with no undesirable odor, making them highly favored by consumers and demonstrating significant economic value and broad development prospects [1]. In intensive fattening production systems, high-energy concentrate feeds are widely used to achieve rapid growth and efficient finishing. Corn, owing to its high starch content and excellent palatability, is the preferred energy source in the diet of meat sheep, and its price has a substantial impact on the economic efficiency of production. Therefore, improving corn utilization efficiency, reducing feed costs, and enhancing production efficiency have become urgent needs to promote cost reduction, efficiency enhancement, and sustainable development in the sheep industry.

Traditionally, conventional ground corn is harvested when the grain moisture content drops below 14% and is primarily preserved through natural drying or mechanical drying for long-term storage. However, this processing method not only consumes high amounts of energy, increasing fuel and equipment costs and significantly raising feed expenses, but also makes the grain susceptible to mold and quality deterioration under improper storage conditions or poor management [2]. In contrast, high-moisture corn (HMC) is a novel, high-energy feed produced by harvesting corn kernels at a moisture content of 25–38% (from the late milk stage to the dough stage), followed by grinding, processing, and sealed storage to undergo anaerobic fermentation. Depending on the raw material composition, HMC can be categorized into different types, such as grain silage and ear corn silage. Its processing primarily involves crushing to disrupt the kernel structure, the addition of organic acids or lactic acid bacteria as silage inoculants to promote anaerobic fermentation, and compaction and sealing for storage [3]. Compared with conventional dry corn, HMC has several nutritional advantages, it effectively preserves nutrients, significantly improves starch digestibility, enhances palatability, inhibits mold growth, and reduces the risk of mycotoxins. Additionally, its relatively higher fiber content helps maintain rumen health while effectively lowering feeding costs [4].

Previous studies under typical total mixed ration systems have shown that feeding HMC can improve production performance and milk fat concentration in dairy cows [5], as well as enhance growth performance, meat yield, and meat quality in beef cattle [6]. In sheep production, under intensive fattening conditions with high-concentrate diets, HMC supplementation has been shown to increase average daily gain in lambs [7,8]. These findings suggest that HMC has the potential to improve production efficiency and product quality while reducing feeding costs.

However, systematic research on its effects in meat-producing rams, particularly in Kazakh sheep, remains limited. Given that HMC undergoes anaerobic fermentation, producing organic acids that can lower ruminal pH and alter the profiles of volatile fatty acids, we hypothesize that its effects extend beyond merely improving growth performance to fundamentally modulating muscle energy metabolism and lipid deposition. Specifically, the increased production of propionate resulting from HMC fermentation may serve as a substrate for de novo fatty acid synthesis, thereby influencing intramuscular fat content and fatty acid composition. Furthermore, because the gut microbiota plays a critical role in nutrient digestion, fermentation, and host metabolic regulation, dietary substitution with HMC is expected to alter the intestinal microbial community. Such microbial changes may, in turn, affect the bioavailability of energy and lipid precursors, ultimately impacting meat quality traits. Therefore, this study aims to systematically investigate the effects of the GS diet on meat production performance and mutton quality in Kazakh rams, and to elucidate the underlying mechanisms that regulate the gut microbiota and mutton quality through integrated muscle metabolomics and gut microbiome analyses. Based on previous findings, we propose the following questions. First, compared with a conventional dry corn diet, the GS diet may positively influence meat production performance and meat quality in Kazakh rams by improving energy utilization efficiency, and may alter the composition of amino acids and fatty acids in muscle. Second, the GS diet may modulate pathways related to muscle energy metabolism, thereby affecting intramuscular fat deposition and fatty acid composition. Third, intake of the GS diet is expected to reshape the gut microbiota composition of Kazakh rams, enriching beneficial bacterial populations while suppressing potential pathogens, thereby mediating effects on meat quality and correlating with changes in the muscle fatty acid profile. Testing these hypotheses will provide a scientific basis for improving meat production performance and quality in Kazakh sheep, reducing feed costs, and promoting the sustainable development of the sheep industry.

## 2. Materials and Methods

This experiment was conducted from October 2024 to March 2025 at a sheep farm in Wigington Town, Hutubi County, Xinjiang. The animal care and experimental procedures involved in this trial were approved by the Animal Welfare Ethics Committee of Xinjiang Agricultural University (Approval No: 20240806, Date of Issue: 19 February 2024).

### 2.1. Experimental Scheme and Animal Feeding

Thirty-two healthy Kazakh rams, approximately six months old with similar body weights (35.4 ± 1.46 kg), were selected as experimental subjects. The animals were randomly assigned to two groups: the control group (CT group) and the experimental group (GS group), with 16 sheep in each group. There was no significant difference in the initial body weight between the two groups as determined by an independent samples *t*-test (*p* > 0.05). The CT group was fed a basal diet containing 24% ordinary crushed corn (on a dry matter basis), while the GS group was fed a basal diet in which half of the ordinary crushed corn was replaced with HMC, resulting in a concentrate containing 12% ordinary crushed corn and 12% HMC. Each group consisted of four replicates, with four sheep per replicate, and sheep within the same replicate were housed together in a single pen.

The HMC and all other feeds used in the experiment were purchased from Xinjiang Tian run Dairy Co., Ltd. (Urumqi, China). After harvesting, the HMC was crushed to a mean particle size of approximately 4–6 mm. Fermentation was carried out using a mixed feed additive supplied by Shanghai Yuanyao Biological Co., Ltd. (Shanghai, China), whose primary microbial community consisted of *Lactobacillus* species (including *Lactococcus lactis* and *Lactobacillus brevis*) at a concentration of 1.3 × 10^11^ CFU/g. The crushed HMC was mixed with the additive, packed tightly, and sealed in silage bags, then stored as wrapped silage in a cool, well-ventilated area for 64 days. Before opening, the pH of the HMC silage was measured as 3.83 ± 0.21. After opening, the HMC was used within the same day, and no mold growth was observed by visual inspection before each feeding. The ambient temperature during the experimental period ranged from –10 °C to 15 °C. Because the trial was conducted in autumn and winter with low ambient temperatures, the temperature of the HMC at feeding was recorded daily and ranged from 4 °C to 8 °C, with no signs of heating or spoilage; therefore, no additional temperature control measures were applied.

The basal diet was formulated according to the NY/T 816-2021 standard [9] for meat sheep with an initial body weight of approximately 35 kg and a target average daily gain of 300 g under intensive fattening conditions. The detailed composition is shown in Table 1. The roughage portion of the diet consisted of cotton residue and whole plant corn silage. The experimental rams were uniformly dewormed and rumen function adjusted before the experiment began. The pre-experiment period lasted 7 days, and the main feeding period was 120 days. During the experiment, feeding was scheduled at 07:00 and 19:00 daily, with free drinking water. All experimental sheep were raised under identical environmental conditions to ensure comparability of the experimental results.

### 2.2. Sample Collection

On the 120th day of the experiment, all the rams were weighed fasting before the morning feeding, and their pre-slaughter live weights were recorded. All rams were subjected to a 24 h feed withdrawal with free access to drinking water prior to slaughter to empty the gastrointestinal tract. After the fasting and dehydration process, the sheep were loaded into vehicles in batches according to their groups, with the loading process kept quiet to minimize stress. Anti-slip bedding was placed at the bottom of the vehicles and good ventilation was ensured. During the transportation, the vehicles were driven smoothly to avoid sudden braking and severe jolts. The transportation time was controlled within 1 h. After the sheep arrived at the slaughterhouse, they underwent health checks. If there were no abnormalities, the sheep were sent to the slaughtering line for stunning and bleeding. The entire procedure complied with the national standard Welfare Criteria for Animals to be Slaughtered (GB/T 42304-2023) [11] for pre-slaughter handling. The carcasses were immediately placed in a 4 °C refrigeration condition for 24 h after bleeding. After the refrigeration, 300 g of the *Longissimus lumborum* muscle samples were collected, packed in cryopreservation tubes, and immediately frozen in liquid nitrogen for subsequent analysis of fatty acids, amino acid composition, and muscle metabolic groups; another 50 g of the LL muscle samples were taken for the determination of meat quality and physical properties. The intestinal contents required for microbiological analysis were also packed in cryopreservation tubes and stored in liquid nitrogen. All the remaining collected samples were stored at −20 °C for long-term storage.

#### 2.2.1. Slaughter Performance Assessment

Pre-slaughter live weight was recorded one day before slaughter using a commercial electronic scale (Model: DTC001-A, Dongmei, Shenzhen, China; accuracy: ±0.05 kg). Carcass weight, head and hoof weight, and tail fat weight were measured after exsanguination. Tail fat weight was weighed immediately after exsanguination, head and hoof weight were measured after removal, and carcass weight was determined after exsanguination, skinning, removal of the head, hooves, and viscera, followed by a 30 min equilibration period. Net meat weight was defined as the total boneless meat obtained after deboning the carcass.

Dressing percentage = (carcass weight/pre-slaughter live weight) × 100

Net meat percentage = (net meat weight/pre-slaughter live weight) × 100

Backfat thickness and ribeye area were measured at the interface of the 12th and 13th ribs, directly above the midpoint of the *Longissimus lumborum* muscle. Backfat thickness was measured using a digital vernier caliper (1058-150C, INSIZE, Shanghai, China), and ribeye area (i.e., the cross-sectional area of the *Longissimus lumborum* muscle) was traced onto transparent paper and subsequently quantified using a transparent acetate grid overlay.

#### 2.2.2. Determination of the Physicochemical Properties of the *Longissimus lumborum*

The ultimate pH (pH_24_) was measured 24 h post-mortem using a meat pH meter (HI981036, HANNA Instruments, Woonsocket, Rhode Island, USA) equipped with a penetrating electrode. The meter was calibrated with standard solutions (pH 4.00 and 7.00) prior to analysis. On the left side of each carcass at the 12th rib level, the electrode was inserted approximately 4 cm into the *Longissimus lumborum* muscle, and measurements were taken in triplicate at five distinct locations. Meat color was evaluated in the CIE L*a*b* color space using a chroma meter (CR-10, Konica Minolta, Tokyo, Japan) calibrated with a white standard plate (Y = 92.8, x = 0.3160, y = 0.3323). Muscle samples were equilibrated at 4 °C for 30 min, and three random measurements were taken to determine lightness (L*), redness (a*), and yellowness (b*). Drip loss was determined following Holman et al. [12]. Trimmed meat samples (50 × 30 × 20 mm; 6 ± 0.50 g) were weighed immediately after trimming to obtain the initial weight, then placed in air-filled, sealed plastic bags and stored at 4 °C for 24 h. After storage, the samples were reweighed to obtain the final weight. Drip loss was calculated as: [(Initial weight − Final weight)/Initial weight] × 100. For sheer force measurement, samples were heated in a thermostatically controlled water bath (DK-8D, Jin Yi Instrument Technology Co., Ltd., Beijing, China) at 80 °C until their internal temperature reached 70 °C, then immediately cooled in cold water to 20 °C. From each *Longissimus lumborum* sample, four parallelepiped subsamples (3–4 cm long, 1 cm wide, 1 cm high) were prepared. Shear force was measured using a tenderness tester (C-LM3B, TENOVO, Beijing, China) set to traverse the samples at 25 cm/min perpendicular to the muscle fiber orientation. Each subsample was measured four times, and the mean value was recorded (expressed in N/cm^2^). Intramuscular fat (IMF) content was determined by Soxhlet extraction. Minced muscle samples were dried at 65 °C in a convection oven (GZX-9246MBE, Jinan, China), ground, and extracted with anhydrous petroleum ether in an automatic fat analyzer (Sox406, Haining, China). IMF content was calculated as (weight of extracted fat/weight of initial dry sample) × 100. Crude protein was quantified using the Kjeldahl method with an automated analyzer (K1100, Haining, China), and nitrogen content was converted to crude protein using a factor of 6.25. For histological analysis, muscle tissue sections were prepared and stained with hematoxylin-eosin (HE) following Fu et al. [13]. At 24 h post-mortem, approximately 1 cm^3^ samples were collected from the core of the *Longissimus lumborum* (12th–13th rib interface) from the left side of eight randomly selected carcasses per group. Samples were immediately fixed in 10% neutral-buffered formalin for 48 h at room temperature, then dehydrated in a graded ethanol series using an automated tissue dehydrator (MTP, SLEE, Nieder-Olm, Germany) and embedded in paraffin wax (KD-BMLLL, Jinhua, China). Transverse sections (5 μm thick) were cut perpendicular to the muscle fibers using a rotary microtome (RM2245, Leica, Nussloch, Germany), floated on a warm water bath, and mounted on glass slides. Following deparaffinization and rehydration, sections were stained with hematoxylin (Service bio, Wuhan, China) for 3 min, rinsed, differentiated, blued, and counterstained with eosin for 30 s. Stained sections were dehydrated, cleared in xylene, and mounted with neutral resin. Whole-slide digital scanning was performed using an automated slide scanner (Lise-Meitner-Str.11-D-55129 Mainz, Germany).

#### 2.2.3. Amino Acid and Medium-to-Long-Chain Fatty Acid Analysis

Amino acid profiling of the Longissimus lumborum muscle was performed for the CT and GS groups. A total of 21 amino acids were analyzed, including the 20 standard proteinogenic amino acids and hydroxyproline. Hydroxyproline is a characteristic amino acid of collagen, and its content is closely associated with meat tenderness; therefore, it was included in the analysis to evaluate the effect of HMC on mutton tenderness. A primary stock solution containing 21 amino acids was prepared in 0.1 M hydrochloric acid, and a mixed internal standard (IS) solution (L-Trp-d5, L-Gln-d5, L-Lys-d4; each at 100 μg/mL) was formulated in 25 mM trichloroacetic acid (TCA). The stock solution was serially diluted with 25 mM TCA to generate ten working standards, each of which was then combined with an equal volume of a 400 ng/mL mixed IS solution to produce calibration samples. For tissue analysis, 20 mg muscle samples were homogenized in water containing 0.15% sodium deoxycholate (DOC), fortified with 4 μL of the IS solution, and sonicated. Proteins were precipitated by adding 10 M TCA and incubating at –20 °C, followed by centrifugation. The supernatant was appropriately diluted and filtered through a 0.2 μm PTFE membrane prior to analysis. Chromatographic separation was achieved using an Advance Bio MS Spent Media column (2.1 × 50 mm, 2.7 μm; Agilent Technologies, Santa Clara, CA, USA) maintained at 40 °C, with mobile phases consisting of (A) water/acetonitrile (95:5, *v*/*v*) containing 0.1% formic acid and 10 mM ammonium format, and (B) acetonitrile/water (95:5, *v*/*v*) containing the same additives. Mass spectrometric analysis was performed on a SCIEX QTRAP 6500+ system (SCIEX, Framingham, MA, USA) operating in scheduled multiple reaction monitoring (MRM) mode with an iron spray voltage of ±5500 V. Data were processed using AB Sciex OS software (2.1, SCIEX, Framingham, MA, USA), with calibration curves constructed by plotting the peak area ratio (analyte to IS) against concentration, and sample concentrations derived from the corresponding regression equation.

Fatty acid profiling was performed for the CT and GS groups. A primary standard stock solution containing 51 fatty acid methyl esters (FAMEs) was prepared in n-hexane (Solution A), which was then diluted tenfold with n-hexane to obtain Solution B. Calibration working standards were subsequently prepared by further diluting Solution B with n-hexane. For sample analysis, 50 mg muscle tissue was homogenized in a dichloromethane/methanol mixture (1:1, *v*/*v*) using a cryogenic grinding mill, followed by sonication and incubation at –20 °C. After centrifugation, the supernatant was collected and evaporated to dryness under nitrogen. The residue was derivatized with 0.5 mol/L methanolic sodium hydroxide at 60 °C and extracted with n-hexane. The upper hexane layer was transferred for GC-MS analysis, performed on an Agilent 8890B-7000D GC-MS system (Agilent Technologies, Santa Clara, CA, USA) equipped with a CP-Sil 88 capillary column (100 m × 0.25 mm × 0.2 μm; Agilent Technologies, Santa Clara, CA, USA). Helium was used as carrier gas at a constant flow rate of 1.0 mL/min, with an injector temperature of 260 °C and a 50:1 split ratio. The oven temperature was programmed as follows: initial 80 °C, increased to 180 °C at 8 °C/min, then to 220 °C at 4 °C/min, and finally to 230 °C at 2 °C/min (held for 13.5 min). Mass spectrometric detection was performed in electron impact (EI) mode using selected ion monitoring (SIM). Chromatographic peaks were processed with Mass Hunter Software (version 10.0, Agilent Technologies, Santa Clara, CA, USA), and concentrations were determined from calibration curves plotting peak area against concentration.

#### 2.2.4. Muscle Metabolomics

For metabolomic profiling, 100 mg of longissimus muscle tissue was combined with 400 μL of methanol (A452-4, Fisher Chemical, Thermo Fisher Scientific, Waltham, MA, USA), vortex-mixed for 1 min, and subjected to five cycles of ultrasonication in an ice-water bath (1 min each, with 1 min intervals). After centrifugation at 14,000× *g* for 10 min at 4 °C using a refrigerated centrifuge (e.g., 5424 R, Eppendorf, Hamburg, Germany), the supernatant was collected and lyophilized overnight in a freeze dryer (e.g., Alpha 1-4 LDplus, Martin Christ, Osterode am Harz, Germany). The dried residue was reconstituted in 80 μL of 50% acetonitrile containing internal standards. Chromatographic separation was performed on a Thermo Vanquish UHPLC system (Thermo Fisher Scientific, Waltham, MA, USA) using a Waters BEH C18 column (1.7 μm, 2.1 × 100 mm; Waters Corporation, Milford, MA, USA) maintained at 40 °C. The mobile phase differed by ionization mode: for positive mode, (A) water with 0.1% formic acid and (B) acetonitrile with 0.1% formic acid; for negative mode, (A) 10 mM ammonium acetate with 0.1% ammonium hydroxide in water and (B) pure acetonitrile. The gradient program was: 98% A (0–1 min), 98–1% A (1–8 min), hold at 1% A (8–10 min), and re-equilibration at 98% A (10.1–12 min). The flow rate was 0.3 mL/min, and the injection volume was 5 μL. Mass spectrometry was conducted on a Thermo QE HF-X instrument (Thermo Fisher Scientific, Waltham, MA, USA) equipped with a heated electrospray ionization (HESI) source. Spray voltages were set to 3500 V (positive mode) and 2500 V (negative mode), with ion transfer tube and vaporizer temperatures at 320 °C and 300 °C, respectively. Sheath and auxiliary gas flows were 30 and 10 arbitrary units. Full MS scans were acquired over *m*/*z* 150–2000 at a resolution of 30,000. Data acquisition and processing were performed using Xcalibur software (4.5, Thermo Fisher Scientific, Waltham, MA, USA) and Compound Discoverer (3.3, Thermo Fisher Scientific, Waltham, MA, USA) for peak alignment, metabolite identification, and statistical analysis. After peak alignment and identification, metabolite features were obtained. A sequential filtering procedure was used to identify differential metabolites between the CT and GS groups, requiring a variable importance in projection (VIP) > 1.0 from the OPLS-DA model, an absolute log2 fold change (|log2FC|) > 0.5, a raw *p*-value < 0.05 from Student’s *t*-test, and a false discovery rate (FDR)-adjusted q-value < 0.05 after Benjamini–Hochberg correction.

#### 2.2.5. Gut Microbiota Analysis

Jejunal content samples collected at slaughter were sent to Kaitai Biotechnology Co., Ltd. (Shanghai, China) for microbiota analysis. Microbial diversity was assessed by amplifying the hypervariable V3-V4 region of the bacterial 16S rRNA gene using barcode-specific primers 338F (5′-ACTCCTACGGGAGGCAGCA-3′) and 806R (5′-GGACTACHVGGGTWTCTAAT-3′) with NEB Q5 High-Fidelity DNA Polymerase (New England Biolabs, Ipswich, MA, USA). The PCR protocol comprised initial denaturation at 98 °C for 30 s, followed by 25–27 cycles of denaturation at 98 °C for 15 s, annealing at 50 °C for 30 s, and extension at 72 °C for 30 s, with a final extension at 72 °C for 5 min. PCR products were quantified using the Quant-it Pico Green dsDNA Assay Kit (Invitrogen, Thermo Fisher Scientific, Waltham, MA, USA) on a microplate reader (FLx800, Bio Tek, Winooski, VT, USA; now part of Agilent Technologies, Santa Clara, CA, USA) and pooled in equimolar ratios according to the required sequencing depth. Sequencing libraries were constructed using the Illumina TruSeq Nano DNA LT Library Prep Kit (Illumina, San Diego, CA, USA), which included DNA end repair, 3′ adenylation, adapter ligation with index sequences, and library amplification, with purification at each stage using BECKMAN AM Pure XP beads (Beckman Coulter, Brea, CA, USA). Library quality was verified on a Lab Chip system (PerkinElmer, Waltham, MA, USA), and qualified libraries were sequenced on an Illumina NovaSeq 6000 platform (Illumina, San Diego, CA, USA) with the NovaSeq 6000 SP Reagent Kit (500 cycles) (Illumina, San Diego, CA, USA), generating 2 × 250 bp paired-end reads. Raw sequencing data were processed using QIIME 2 (version 2022.2, https://qiime2.org) for quality filtering, denoising, and downstream analysis.

### 2.3. Data Analysis

All statistical analyses were performed with the pen as the experimental unit, as animals within the same pen were not independent due to shared housing and feeding conditions. For each pen, the values of the four rams were averaged to obtain a single pen mean for each measured trait (e.g., slaughter performance, meat quality, fatty acid and amino acid contents). Thus, each treatment group had *n* = 4 pen replicates (4 pens per group, 4 animals per pen).

#### 2.3.1. Sample Size Adequacy

A priori power analysis was performed based on the primary outcome—muscle shear force. According to preliminary results, the expected difference in shear force between GS and CT groups was 22.4 N/cm^2^, with a pooled standard deviation of 5.3 N/cm^2^, yielding a Cohen ‘s d of approximately 4.2. Assuming a two-sided independent samples *t*-test, α = 0.05, and power (1-β) = 0.80, the required number of pens per group was calculated to be 2. In the present study, we used 4 pens per group (*n* = 4 pen replicates, 4 animals per pen), achieving a power > 0.95 for the primary outcome. For metabolomics and microbiota analyses, sample sizes were determined based on previous similar studies; these analyses are exploratory, and false discovery rate (FDR) correction was applied to control type I error.

#### 2.3.2. Slaughter Performance, Meat Physicochemical Properties, and Muscle Amino Acid and Fatty Acid Composition

Data were based on pen means (*n* = 4 per group). Independent samples *t*-tests were performed using SPSS 27.0 (IBM Corp., Armonk, NY, USA) to compare differences between the CT and GS groups. Prior to the *t*-tests, normality was assessed using the Shapiro–Wilk test, and homogeneity of variances was checked using Levene’s test.

#### 2.3.3. Muscle Metabolomics

Four rams were selected from the CT group (4 pens, one ram per pen), and eight rams were selected from the GS group (4 pens, two rams per pen). Four rams were selected from the CT group (4 pens, one ram per pen), and eight rams were selected from the GS group (4 pens, two rams per pen). This unbalanced design was chosen to increase the statistical power for detecting differential metabolites within the GS group, which was the primary focus of the metabolomics analysis, while maintaining representation from the CT group. Principal component analysis (PCA) and orthogonal partial least squares-discriminant analysis (OPLS-DA) were first performed using SIMCA 14.1 software (Sartorius Stedim Data Analytics AB, Umea, Sweden) to evaluate the separation trend of metabolite profiles between the two groups. Differentially expressed metabolites were then identified using Student’s t-test combined with fold change analysis, with screening criteria set as variable importance in projection (VIP) > 1 (derived from the OPLS-DA model) and raw *p* < 0.05. To control for multiple comparisons, false discovery rate (FDR) correction was applied using the Benjamini–Hochberg method, and a q-value < 0.05 was considered statistically significant. FDR correction was performed using R software version 4.2.2 (R Foundation for Statistical Computing, Vienna, Austria).

#### 2.3.4. Gut Microbiota Analysis

Eight rams were selected from each group (4 pens per group, two rams per pen). Data processing was carried out using QIIME 2 (version 2022.2). Alpha diversity indices (ACE, Chao1, Shannon, Simpson) were calculated, and principal coordinate analysis (PCoA) based on Bray- Curti’s distance was performed to assess microbial community diversity and structural differences. Permutational multivariate analysis of variance (PERMANOVA) with 999 permutations was used to test differences in beta diversity between the two groups. Differentially abundant bacterial taxa were identified using LEfSe analysis (online Galaxy plat for), with screening criteria set as LDA score > 2 and *p* < 0.05. Where appropriate, FDR correction was applied for multiple comparisons in the microbiota data.

#### 2.3.5. Correlation Analysis Between Gut Microbiota and Fatty Acids

Spearman’s rank correlation coefficient was used to assess the associations between the relative abundances of bacterial phyla and the content of individual fatty acids in the longissimus muscle. Spearman’s correlation was chosen because the data were not normally distributed. Correlation analyses were performed using R software (version 4.2.2). To control for the false discovery rate (FDR) due to multiple comparisons, the Benjamini–Hochberg method was applied to adjust *p*-values. An adjusted *p*-value (q-value) < 0.05 was considered statistically significant. Both the correlation coefficients (ρ) and the adjusted q-values are presented in the heatmap.

All results are presented as mean ± standard deviation (SD) of pen means, unless otherwise stated. Exact *p*-values are reported where possible. For all statistical tests, *p* < 0.05 was considered statistically significant, and *p* < 0.01 was considered highly significant.

## 3. Results

### 3.1. Effects of Feeding HMC on the Meat Production Performance of Kazakh Rams

The results of the effect of feeding HMC on the meat production performance of Kazakh rams are shown in Table 2. There was no significant difference in live weight before slaughter between the two groups of Kazakh rams (*p* > 0.05). The backfat thickness of the GS group was significantly higher than that of the CT group (*p* < 0.05). There were no statistically significant differences in carcass weight, net meat weight, head and hoof weight, tail fat weight, dressing percentage, net meat percentage, or ribeye area in the GS group (*p* > 0.05).

### 3.2. Effects of Feeding HMC on the Mutton Quality of Kazakh Rams

#### Effects of HMC on Physicochemical Properties of Mutton from Kazakh Rams

**Figure 1:**
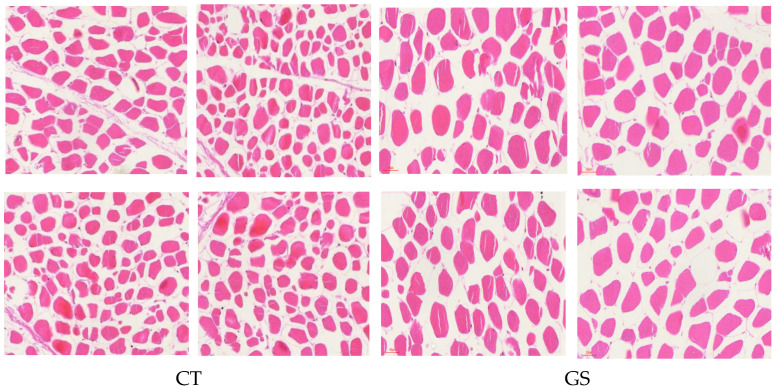
Representative H&E-stained cross-sections of the *Longissimus lumborum* muscle. A vertical line separates the CT and GS images. Scale bar = 20 μm. Images are representative of 4 rams per group (one ram per pen). H&E, hematoxylin and eosin. Qualitative assessment visually suggests greater adipocyte accumulation in the GS group.

The effects of HMC feeding on the physicochemical properties of mutton from Kazakh rams are summarized in Table 3 and Figure 1. Muscle shear force was significantly lower (*p* < 0.01) in the GS group compared to the CT group. Intramuscular fat content showed a 56.34% increase (*p* < 0.01) in GS-fed rams relative to controls. No significant differences (*p* > 0.05) were observed between groups in pH 24h, meat color, or drip loss. Histological examination visually suggested greater intramuscular fat deposition in the GS group compared with the CT group (Figure 1).

### 3.3. Effects on Muscle Amino Acid and Fatty Acid Content

#### 3.3.1. Effects of Feeding HMC on the Amino Acid Content in the Muscle of Kazakh Rams

The results showing the impact of feeding HMC on the muscle amino acids of Kazakh rams are presented in Table 4. Among the 21 amino acids, the aspartic acid content in the CT group was significantly higher than that in the GS group (*p* < 0.01). The arginine and glutamine contents in the CT group were significantly higher than those in the GS group (*p* < 0.05). The glycine content in the CT group was significantly lower than in the GS group (*p* < 0.05). There were no differences in the contents of 17 amino acids including alanine, asparagine, and glutamic acid (*p* > 0.05).

#### 3.3.2. Effects of Feeding HMC on the Fatty Acid Content in the Muscle of Kazakh Rams

The results showing the effects of feeding HMC on the muscle fatty acids of Kazakh rams are presented in Table 5. The contents of Methyl undecanoate, Methyl myristate, Methyl palmitate, Methyl heptadecanoate, Methyl alpha linoleate, and All-cis-4,7,10,13,16-docosapentaenoic acid in the GS group were significantly higher than those in the CT group (*p* < 0.01). The contents of Methyl caprate, Methyl laurate, Methyl Tri decanoate, Methyl pentadecanoate, Methyl palmitoleate, Methyl stearate, Methyl transvaccenate, Methyl oleate, Methyl vaccinate, and Methyl docosatetraenoate were significantly higher in the GS group than in the CT group (*p* < 0.05). There were no differences in the contents of fatty acids such as Methyl caproate, Methyl caprylate, and Methyl myristylation between the two groups (*p* > 0.05).

### 3.4. Effects of Feeding HMC on Muscle Metabolomics in Kazakh Rams

#### 3.4.1. Principal Component Analysis

**Figure 2:**
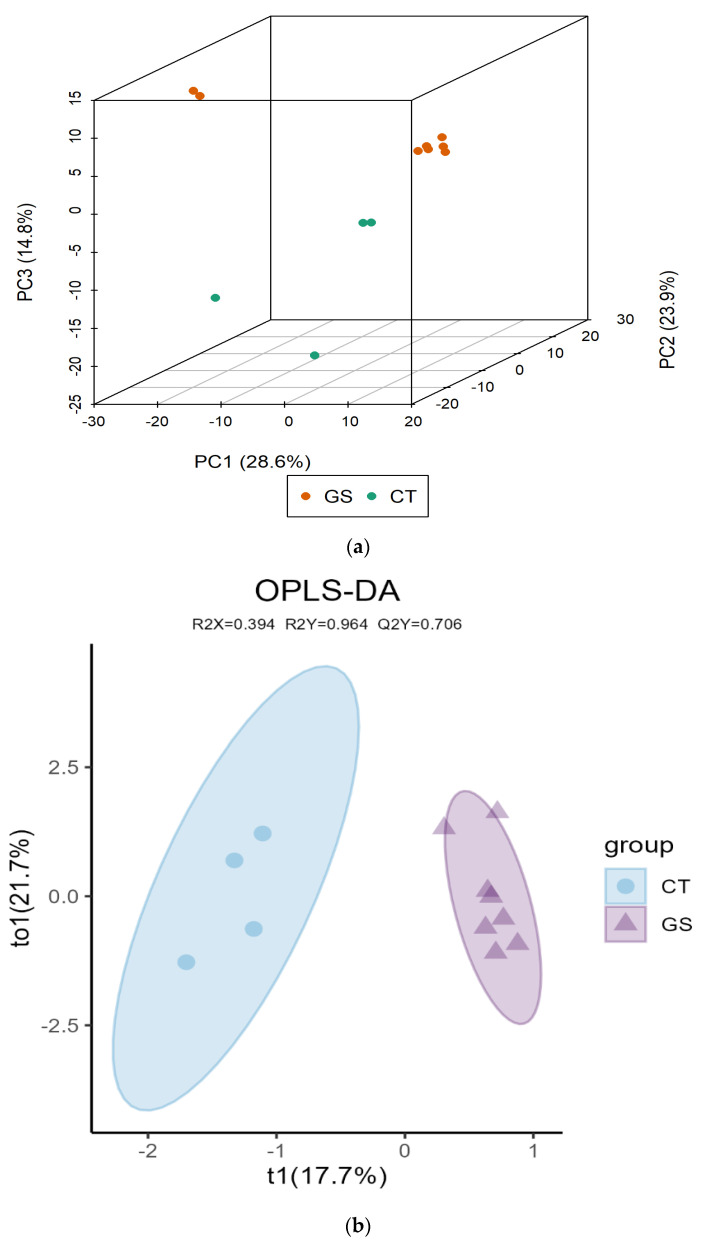

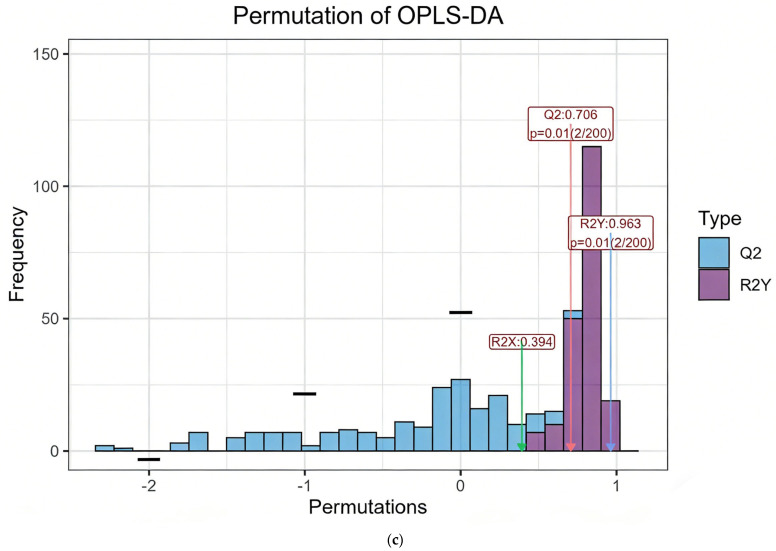
Principal component analysis diagram between the two groups. (**a**) Three-dimensional PCA score plot. PC1, PC2, and PC3 represent the first three principal components, explaining 28.6%, 23.9%, and 14.8% of the total variance, respectively. Each sphere represents an individual muscle sample; red spheres = GS group (*n* = 8), blue spheres = CT group (*n* = 4). The spatial separation between groups indicates distinct metabolic profiles; (**b**) Orthogonal partial least squares-discriminant analysis (OPLS-DA) score plot of muscle metabolomics data between GS group and CT group. The horizontal axis (t [1]) represents the predictive principal component (explaining 17.7% of variance), capturing the maximum separation between groups. The vertical axis (to [1]) represents the orthogonal principal component (explaining 21.7% of variance), capturing within-group variation. GS samples (red) cluster at approximately (0.8, –0.2), and CT samples (blue) cluster at approximately (–0.8, 0.2). Model quality parameters: R^2^Y = 0.963, Q^2^ = 0.706.; (**c**) The OPLS-DA model, validation plot displays the horizontal axis representing model accuracy and the vertical axis showing the frequency of classification outcomes. Specifically, this model conducted 200 randomized permutation experiments on datasets. When Q2’s *p*-value reaches 0.01, it indicates that 4 randomized grouping models outperformed the OPLS-DA model in this permutation test. If R2Y’s *p*-value equals 0.545, it suggests that 109 randomized grouping models demonstrated higher explanatory power for the Y matrix compared to the OPLS-DA model. Generally, models with *p*-values below 0.05 are considered optimal.

To increase the power for detecting differential metabolites, eight rams from the GS group (two per pen) and four rams from the CT group (one per pen) were selected for muscle metabolomics analysis, based on their representativeness within each pen. Principal component analysis results are presented in Figure 2. The principal component analysis (PCA) score plot (PC1 vs. PC2) showed significant differences in metabolite distribution between the muscle samples of the Kazakh rams in the CT and GS groups under both positive and negative ion modes (2a). The orthogonal partial least squares-discriminant analysis (OPLS-DA) score plot clearly showed significant distinction and separation between the GS and CT groups (2b), indicating that the GS diet significantly affects muscle metabolism in Kazakh rams. The OPLS-DA model was validated by a permutation test (200 random permutations). As shown in Figure 2c, the R^2^Y value (0.963) and Q^2^ value (0.706) of the original model were both significantly higher than those of the permuted models, with the Q^2^ intercept being < 0.05 (*p* = 0.01). These results indicate that the OPLS-DA model had good predictive ability and no overfitting, confirming the reliability of the differential metabolite screening.

#### 3.4.2. Screening and Analysis of Differential Metabolites

**Figure 3:**
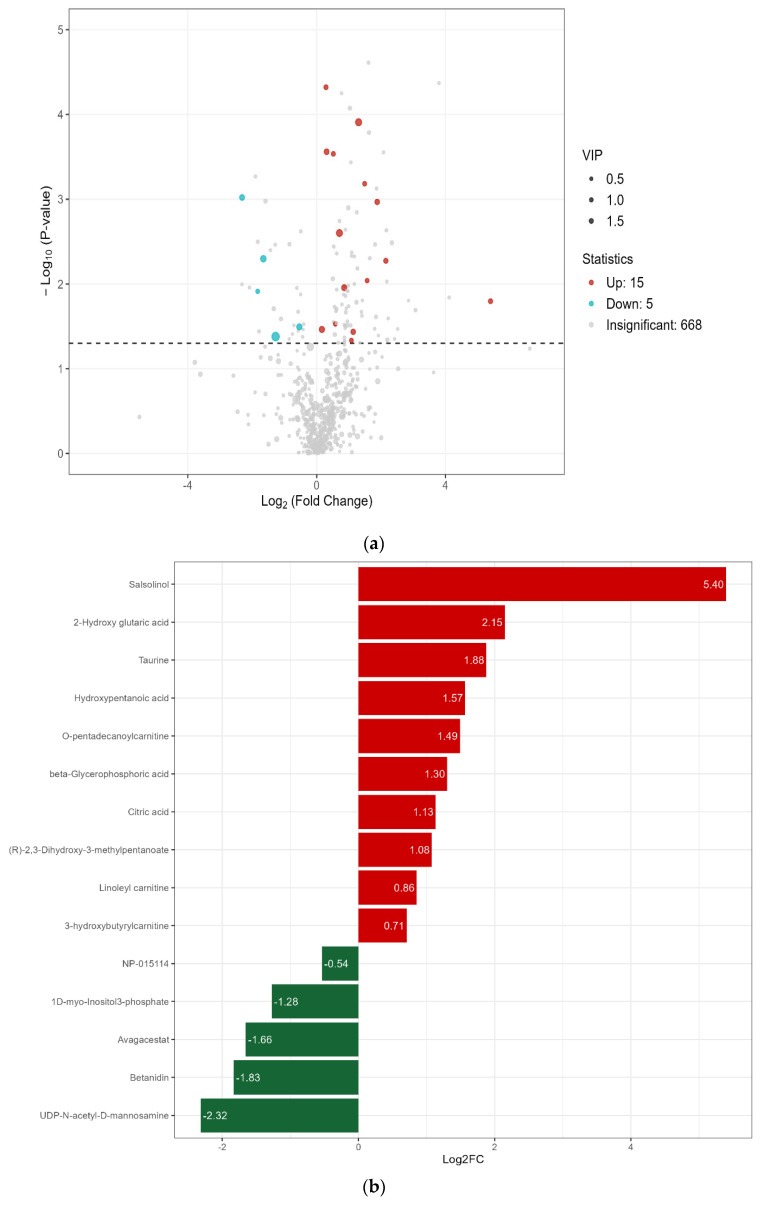
Differential metabolite analysis between GS and CT groups. (**a**) Volcano plot. Each point represents a metabolite. Blue: significantly downregulated; red: significantly upregulated; gray: not significantly different. Horizontal axis: log_2_ fold change (log_2_FC). Vertical axis: –log_10_(*p*-value). Dot size: variable importance in projection (VIP) value from the OPLS-DA model. Significance criteria: VIP > 1, |log_2_FC| > 0.5, raw *p* < 0.05, and FDR-adjusted q < 0.05; (**b**) Bar chart of log_2_FC for the 20 differential metabolites. Red bars: upregulated in GS; green bars: downregulated in GS. Metabolite names are listed on the vertical axis.

The volcano plot visually presents the overall distribution of all detected metabolites (Figure 3a). A total of 668 metabolites were identified from the 12 analyzed samples. Using sequential filtering criteria (VIP > 1.0, |log2FC| > 0.5, raw *p* < 0.05, and FDR-adjusted q < 0.05), 20 metabolites were identified as significantly differential between the CT and GS groups, of which 15 were up-regulated and 5 were down-regulated in the GS group compared with the CT group (Figure 3b).

#### 3.4.3. KEGG Pathway Enrichment Analysis of Differential Metabolites

**Figure 4:**
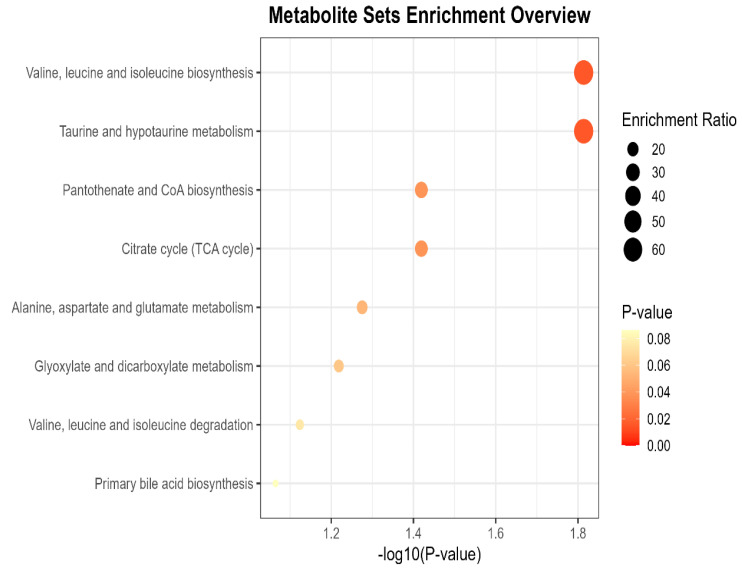
KEGG enrichment impact factor bubble chart for differential metabolites in GS_vs_CT group. Horizontal axis: Rich Factor (ratio of differential metabolites to total metabolites in a pathway). Vertical axis: pathway name (sorted by *p*-value). Point color: *p*-value (redder = more significant). Point size: number of differential metabolites enriched in the pathway.

The KEGG enrichment analysis of differential metabolites showed that the muscle tissue differential metabolites of the two groups were mainly enriched in 8 KEGG metabolic pathways. Among these, four pathways were significantly enriched: Valine, Leucine and Isoleucine Biosynthesis; Taurine and hypo taurine metabolism; Pantothenate and CoA biosynthesis; Citrate cycle (TCA cycle) (Figure 4).

### 3.5. Effects of Feeding HMC on Gut Microbiota Diversity in Kazakh Rams

#### 3.5.1. Alpha Diversity Analysis

The effects of feeding HMC on the gut microbiota alpha diversity of Kazakh rams are shown in Table 6. The ACE, Chao1, Shannon, and Simpson indices in the CT group were 12.96%, 12.86%, 8.56%, and 3.23% higher than those in the GS group, respectively, with no significant differences observed (*p* > 0.05).

#### 3.5.2. Beta Diversity Analysis

**Figure 5:**
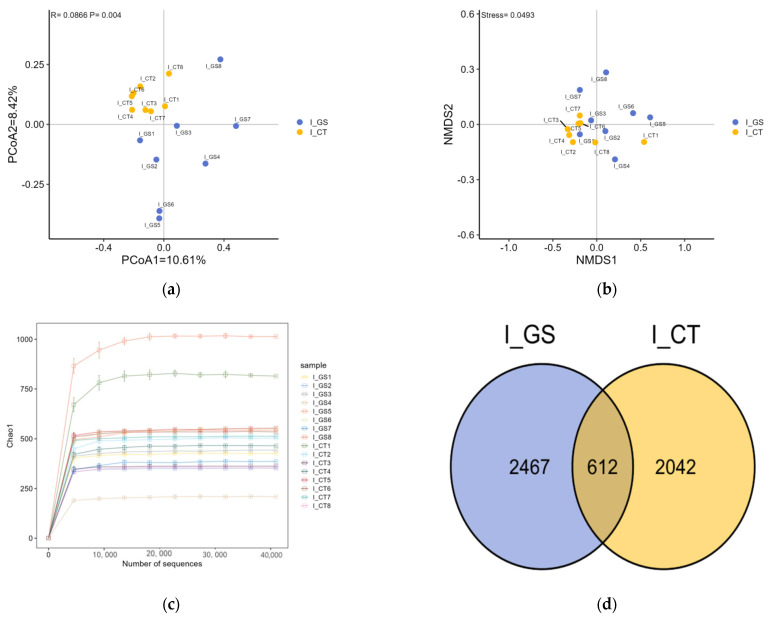
Effects of feeding HMC on gut microbiota Beta diversity in Kazakh rams. (**a**) Principal coordinate analysis (PCoA) based on Bray-Curti’s distance. Each point represents an individual sample; blue = CT group, red = GS group. Horizontal axis: first principal coordinate (PCo1) with percentage indicating its contribution to sample variance; vertical axis: second principal coordinate (PCo2) with percentage. PERMANOVA *p*-value (<0.01) indicates significant separation between groups; (**b**) Non-metric multidimensional scaling (NMDS) analysis. Points of different colors or shapes represent sample groups under different environments or conditions. The scales of the horizontal and vertical axes are the projected coordinates of the sample points on the two-dimensional plane, respectively. It is generally believed that Stress < 0.2 indicates a meaningful ordination; when Stress is less than 0.1, it can be considered a good ranking; when Stress is less than 0.05, it is very representative; (**c**) Rank-abundance curve. Horizontal axis: OTU (operational taxonomic unit) rank ordered by abundance; vertical axis: log_10_ of relative abundance of each OTU. Each line represents a group (CT or GS). A flatter curve indicates higher evenness of community composition; (**d**) Venn diagram showing unique and shared OTUs between groups. Center: 612 OTUs shared; left circle (CT): 2042 unique OTUs; right circle (GS): 2467 unique OTUs. OTUs were clustered at 97% sequence similarity.

Beta diversity analysis revealed a significant separation in microbial community composition between the CT and GS groups, as shown by PCoA (Figure 5a, PERMANOVA, *p* < 0.01) and NMDS (Figure 5b, *p* < 0.05). The Chao1 index dilution curves for the gut samples of Kazakh rams tended to saturate with increasing sequencing depth, indicating adequate coverage of microbial species (Figure 5c). Based on the optimized sequences, the GS and CT groups had 2467 and 2042 unique OTUs, respectively, with a total of 5121 OTUs obtained, of which only 612 were common OTUs (12.0%), indicating significant differentiation in the microbial community structure between the two groups (Jaccard index = 0.12) (Figure 5d).

#### 3.5.3. Species Distribution Analysis at Phylum and Genus Level

**Figure 6:**
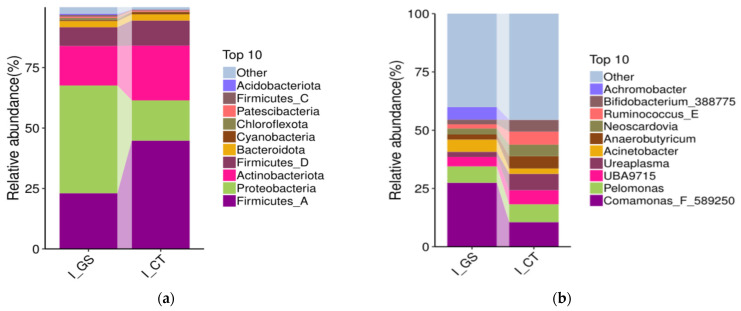
Species abundance of gut microbiota in Kazakh rams feed HMC at the (**a**) phylum and (**b**) genus level, the *x*-axis represents sample names; the *y*-axis represents the relative abundance of species annotated to a certain type.

At the phylum level, the samples were mainly composed of 10 phyla: Acidobacteriota, Firmicutes_C, Firmicutes_D, Firmicutes_A, Patescibacteria, Chloroflexota, Cyanobacteria, Bacteroidota, Actinobacteriota, and Proteobacteria (Figure 6a). Among these, Firmicutes_A, Proteobacteria, Actinobacteriota, and Firmicutes_D were the dominant phyla across all samples. Further analysis revealed that the relative abundances of Acidobacteriota and Proteobacteria were significantly higher in the GS group than in the CT group (*p* < 0.05), whereas the abundance of Firmicutes_A was significantly higher in the CT group (*p* < 0.01).

At the genus level, the samples were primarily composed of ten bacterial genera (Figure 6b). Achromobacter, Bifidobacterium_388775, Ruminococcus_E, Neoscardovia, Anaerobutyricum, Acinetobacter, Ureaplasma, UBA9715, Pelomonas, and Comamonas_F_589250. Among these, Comamonas_F_589250, Pelomonas, and UBA9715 were dominant. The abundances of Ureaplasma, Anaerobutyricum, and Bifidobacterium_388775 were significantly higher in the CT group compared to the GS group (*p* < 0.05), while the abundances of Comamonas_F_589250 and Achromobacter were significantly higher in the GS group compared to the CT group (*p* < 0.01).

#### 3.5.4. LEfSe Analyse

**Figure 7:**
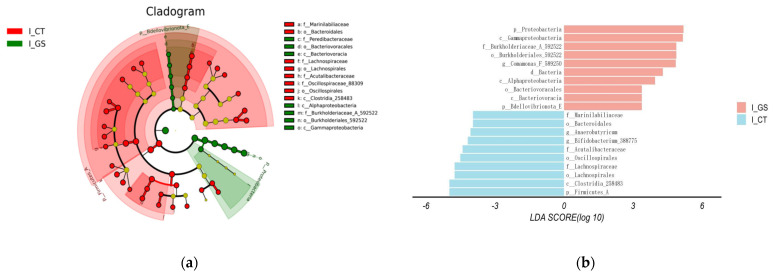
LEfSe Analysis of the Gut Microbial Community in Kazakh Rams Feed HMC. (**a**) Cladogram showing phylogenetic distribution of differentially abundant taxa. Circles from inner to outer rings represent taxonomic levels from phylum to genus. Red: taxa enriched in GS group; green: taxa enriched in CT group; yellow: no significant difference; (**b**) Histogram of linear discriminant analysis (LDA) scores for differentially abundant taxa (LDA score > 2.0). Red bars: higher abundance in GS; green bars: higher abundance in CT.

LEfSe analysis identified 10 bacterial taxa with LDA scores > 2 that were significantly different in abundance between the CT and GS groups (Figure 7). In the GS group, five taxa showed significantly higher abundance compared with the CT group (*p* < 0.05): phylum *Proteobacteria*, class *Gammaproteobacteria*, family *Burkholderiaceae_A592522*, order *Burkholderiales_592522*, and genus *Comamonas_589250*. Conversely, four taxa were significantly lower in the GS group (*p* < 0.05): phylum *Firmicutes_A*, class *Clostridia_258483*, order *Lachnospirales*, and family *Lachnospiraceae*. These differentially abundant taxa are considered key drivers of the beta diversity shift observed between the two groups. While HMC feeding significantly altered the beta diversity of the gut microbiota, it did not affect alpha diversity. Therefore, the microbial modulation induced by HMC is characterized by selective enrichment of specific bacterial lineages rather than a global change in community diversity.

### 3.6. Correlation Analysis Between Gut Microbiota at the Phylum Level and Intramuscular Fatty Acid Content in Kazakh Rams

**Figure 8:**
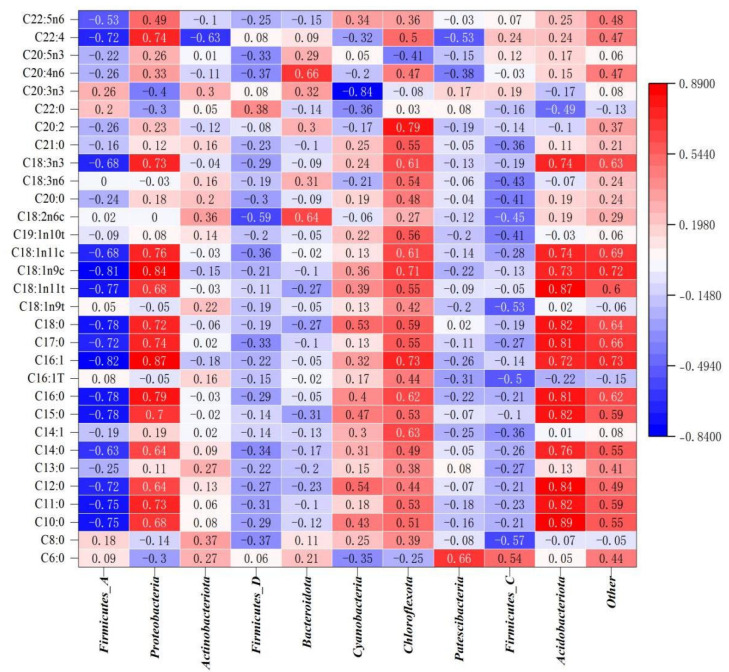
Spearman’s rank correlation heatmap between phylum-level gut microbiota (rows) and intramuscular fatty acid content (rows). Correlation coefficients (ρ) are color-coded: red = positive correlation, blue = negative correlation. Only correlations with FDR-adjusted q < 0.05 are shown. *p*-values were adjusted for multiple comparisons using the Benjamini–Hochberg false discovery rate (FDR) method. The color scale ranges from –1 to +1.

In the correlation analysis (Figure 8), correlation analysis at the phylum level revealed that *Firmicutes-A* was significantly negatively correlated with fatty acids such as Methyl docosatetraenoate, Methyl alpha linolenate, Methyl vaccenate, Methyl oleate, Methyl transvaccenate, Methyl stearate, Methyl heptadecanoate, Methyl palmitoleate, Methyl palmitate, Methyl pentadecanoate, Methyl laurate, Methyl undecanoate, and Methyl caprate (*p* < 0.05). *Proteobacteria* was significantly positively correlated with fatty acids such as Methyl docosatetraenoate, Methyl vaccenate, and Methyl palmitoleate (*p* < 0.01), and significantly positively correlated with fatty acids such as Methyl alpha linolenate, Methyl oleate, Methyl heptadecanoate, Methyl palmitate, and Methyl undecanoate (*p* < 0.05). *Actinobacteriota* was significantly negatively correlated with Methyl docosatetraenoate (*p* < 0.05). *Bacteroidota* was significantly positively correlated with fatty acids such as Methyl linoleate and Methyl arachidonate (*p* < 0.05). *Cyanobacteria* were significantly negatively correlated with Methyl 11-14-17 eicosatrienoate (*p* < 0.05). *Chloroflexota* was significantly positively correlated with fatty acids such as Methyl myristoleate, Methyl palmitoleate, Methyl oleate, and Methyl 11-14 eicosadienoate (*p* < 0.05). *Patescibacteria* was significantly positively correlated with Methyl caproate (*p* < 0.05). *Acidobacteriota* was significantly positively correlated with fatty acids such as Methyl caprate, Methyl undecanoate, and Methyl heptadecanoate (*p* < 0.05).

## 4. Discussion

### 4.1. Effects of Feeding HMC on the Meat Production Performance of Kazakh Rams

In this study, feeding GS group significantly increased backfat thickness in Kazakh rams, but no significant differences were observed in other slaughter performance traits. This indicates that under the current experimental conditions, HMC primarily promotes fat deposition rather than overall growth or carcass yield. The increase in backfat thickness may be attributed to the higher energy utilization efficiency of HMC, the lactic acid produced during its fermentation can lower feed pH, improve ruminal starch digestibility and volatile fatty acid production, thereby directing more energy toward adipose tissue deposition [14]. Similar observations have been reported in finishing beef cattle fed HMC, where backfat thickness increased without concomitant changes in carcass weight [15,16]. However, a study in lambs showed that HMC supplementation increased average daily gain [8], suggesting that responses may vary with species, breed, diet composition, and feeding duration. The lack of significant effects on carcass weight and dressing percentage in the present study may be related to the relatively short feeding period of 120 days, as well as the possibility that under low winter temperatures, the rams may have allocated more energy to maintaining body temperature rather than growth. Furthermore, replacing only 50% of ordinary corn with HMC may not have provided a sufficient increase in dietary energy to alter overall carcass traits. Nevertheless, the selective increase in backfat thickness may still positively affect meat quality, as subcutaneous fat is closely associated with improved juiciness and flavor.

### 4.2. Effects of Feeding HMC on the Mutton Quality of Kazakh Rams

The GS group showed significantly reduced muscle shear force and increased intramuscular fat (IMF) content, indicating improved meat tenderness and enhanced intramuscular fat deposition. The marked decrease in shear force reflects a substantial improvement in tenderness, which is one of the most critical palatability attributes for consumers. The concurrent increase in IMF content is consistent with the well-established inverse relationship between IMF and shear force, as intramuscular adipocytes can physically disrupt muscle fiber bundles and increase intermyofibrillar space, thereby reducing resistance during chewing [17,18]. Histological examination qualitatively revealed a higher density of adipocytes within the longissimus muscle of the GS group (Figure 1), which aligns with the chemically measured increase in IMF, although qualitative observation alone does not constitute quantitative validation. No significant differences in pH_24_h or drip loss were observed between the two groups, indicating that HMC feeding did not adversely affect postmortem glycolysis or water-holding capacity, both of which are important for meat processing and shelf life. The improvements in tenderness and IMF content may be attributed to the higher energy density of the HMC-containing diet. Previous studies have shown that high-moisture corn can increase starch digestibility and ruminal propionate production, thereby enhancing de novo fatty acid synthesis in adipose tissue and muscle [14,19]. Similar effects of HMC feeding on increased milk fat percentage in dairy cows and fat deposition in beef cattle have also been reported [20]. However, the magnitude of IMF increase observed in the present study is more pronounced than in previous sheep studies, which may be related to the breed characteristics of Kazakh sheep or the fact that the animals were at a growth stage sensitive to nutritional regulation. Although HMC improved tenderness and fat content, it did not significantly affect meat color or drip loss, suggesting that the observed changes primarily involve lipid metabolism pathways rather than overall meat quality deterioration. In conclusion, incorporating HMC into the diet of fattening rams effectively enhances two key quality attributes—tenderness and intramuscular fat—without compromising other physicochemical properties.

### 4.3. Effects on Muscle Amino Acid and Fatty Acid Content

In this study, although the contents of most amino acids did not change significantly, the levels of aspartic acid and arginine were significantly decreased while glycine was significantly increased in the GS group. The decreases in aspartic acid and arginine may indicate altered nitrogen metabolism. It has been suggested that the lower pH and higher protein degradability of high-moisture corn can improve ruminal ammonia-N utilization, thereby reduce nitrogen loss and affect amino acid deposition. Glycine, a major component of collagen, may influence meat tenderness through its association with connective tissue properties [21]. Touno et al. [22] found that HMC increased ruminal degradability of dry matter and crude protein in ruminants, while Gallo et al. [23] reported that HMC significantly reduced milk protein content in dairy cows compared with dry corn. These cross-species studies were conducted under high-concentrate or intensive feeding conditions, making them reasonably comparable to the present study. Taken together, Feeding HMC may regulate ruminal nitrogen metabolism and reduce muscle protein deposition, thereby affecting the levels of certain amino acids, although its impact on the overall amino acid profile is limited.

The composition and content of intramuscular fatty acids (IMFAs) are key determinants of meat tenderness, water-holding capacity, and flavor [24]. In the present study, the GS group showed significantly increased levels of various saturated and polyunsaturated fatty acids, including methyl oleate, methyl α-linolenate, and methyl docosapentaenoate. Methyl oleate, the predominant monounsaturated fatty acid, contributes to improved flavor and has potential health benefits such as cholesterol reduction and anti-inflammatory effects [25]; α-linolenic acid and docosapentaenoic acid, as n-3 and n-6 polyunsaturated fatty acids, enhance the nutritional value of lamb [26]. Mechanistically, the higher starch fermentability of HMC may increase ruminal propionate production, providing substrates for de novo lipogenesis. At the same time, HMC may alter ruminal biohydrogenation, reducing the saturation of unsaturated fatty acids and allowing more unsaturated fatty acids to escape ruminal degradation and be deposited in muscle. Silva et al. [27], in a study of beef cattle fed high-concentrate finishing diets containing HMC, also observed increased fatty acid content and an elevated polyunsaturated/saturated fatty acid ratio in the longissimus muscle, consistent with our findings. In summary, HMC feeding not only promotes intramuscular fat deposition but also optimizes fatty acid composition, thereby contributing to improved meat quality in lamb.

### 4.4. Effects of Feeding HMC on Muscle Metabolomics in Kazakh Rams

Through non-targeted metabolomics analysis, this study systematically revealed, for the first time, the effects of HMC feeding on muscle metabolism in Kazakh rams. A total of 668 differential metabolites and their enriched pathways were identified, indicating that HMC significantly influences energy metabolism, lipid synthesis, and antioxidant status by regulating key metabolic nodes, thereby providing a molecular basis for the observed improvement in meat quality. The significantly enriched pathways directly associated with meat quality improvement included the tricarboxylic acid (TCA) cycle, Valine, Leucine and Isoleucine Biosynthesis, taurine and hypo taurine metabolism, and pantothenate and coenzyme A (CoA) biosynthesis.

Upregulation of the TCA cycle indicated that muscle cells in the GS group were in an active state of oxidative metabolism. Citrate, an intermediate of the TCA cycle, can be transported to the cytosol and cleaved by ATP-citrate lyase to generate acetyl-CoA, the direct substrate for de novo fatty acid synthesis. In the present study, the GS group showed a 56% increase in intramuscular fat (IMF) content, along with significantly higher levels of Methyl palmitate, Methyl stearate, and Methyl oleate, which is consistent with the increased acetyl-CoA supply derived from an enhanced TCA cycle. The upregulation of Beta-Glycerophosphoric not only reflected active energy metabolism but also suggested enhanced cell membrane phospholipid metabolism, a key cellular process for IMF deposition. Beta-Glycerophosphoric is an intermediate of glycolysis and the pentose phosphate pathway, and its accumulation further indicates enhanced phospholipid metabolism, closely associated with IMF deposition. Chen et al. [28] found that Beta-Glycerophosphoric promotes collagen deposition, which may be related to the improvement in muscle tenderness observed in this study.

Taurine, a conditionally essential amino acid, is an important antioxidant and cell membrane stabilizer in animals [29]. The enrichment of the taurine metabolism pathway in this study suggests enhanced antioxidant capacity in the muscle of the GS group, which could reduce postmortem lipid peroxidation and protein oxidation, thereby protecting meat color, tenderness, and water-holding capacity. More importantly, taurine has been shown to promote fatty acid uptake and oxidation in muscle. Fan et al. [30] demonstrated that taurine reduces ruminal microbial crude protein synthesis in beef steers by enriching pathways related to sulfur and amino acid metabolism. Dai et al. [31] reported that dietary taurine supplementation promotes growth performance and significantly improves antioxidant capacity and immunity in goats. Studies by Lu, L et al. [32] demonstrated that taurine supplementation can improve muscle mass and promote the transformation of muscle fibers from glycolytic fibers to oxidative fibers. Furthermore, Chen et al. [33] found that taurine significantly increases the content of various polyunsaturated fatty acids in muscle, thereby improving muscle quality, while Ma et al. [34] confirmed that the taurine and hypo taurine metabolic pathway is a key pathway affecting the flavor characteristics of Wuding chicken meat. The marked increase in Methyl alpha-linoleate and All-cis-4,7,10,13,16-docosapentaenoic acid in the GS group may be related to taurine-mediated regulation of fatty acid metabolism.

Acylcarnitine’s are small-molecule metabolites produced during fatty acid oxidative metabolism, and their accumulation generally reflects the dynamic balance between fatty acid Beta-oxidation and synthesis. Huang et al. [35] found in a study on the effects of dietary energy level on plasma and milk metabolism in dairy cows that an increase in acylcarnitine’s contributes to fatty acid synthesis, thereby enhancing milk fat synthesis. Pentadecanoylcarnitine, an odd-chain saturated fatty acid, has been demonstrated to be associated with meat quality in ruminants [36]. In ruminant production, increased muscle content of pentadecanoylcarnitine promotes fatty acid metabolism and thereby improves meat quality [37]. In this study, this metabolite was upregulated in the GS group, consistent with the increased IMF content and enrichment of beneficial fatty acids.

UDP-N-acetyl-O-monoamine was a significantly downregulated differential metabolite. As a very important nucleotide sugar, it serves as a sugar donor for glycosylation reactions in vivo, affecting starch and cell wall biosynthesis. In mammals, protein glycosylation can influence bioactivity, stability, and immunogenicity [38]. Studies have shown that protein glycosylation reduces the biological activity and immunity of mammalian cells [39]. The downregulation of this metabolite may reduce unnecessary glycosylation modifications, thereby helping muscle cells maintain higher metabolic activity and indirectly promoting fat deposition.

Valine, leucine, and isoleucine, as branched-chain amino acids (BCAAs), play important roles in energy metabolism. Qiu et al. [40] found that BCAAs can increase the proportion of polyunsaturated fatty acids through the fatty acid synthesis pathway. Naderi et al. [41] demonstrated that taurine and hypo taurine play key roles in the body’s antioxidant defense and anti-inflammatory responses. BCAAs not only provide carbon skeletons for fat synthesis, isoleucine and valine are catabolized to succinyl-CoA and acetyl-CoA, which enter the TCA cycle, but also directly activate the mTOR signaling pathway to promote adipogenesis. The upregulation of the Valine, Leucine and Isoleucine Biosynthesis pathway in this study coincides with the increase in IMF content and changes in polyunsaturated fatty acid proportions.

Pantothenate and CoA biosynthesis is one of the important pathways of energy metabolism and is involved in fatty acid anabolism. Pantothenate is a precursor of CoA, an essential cofactor for fatty acid activation and mitochondrial Beta-oxidation. The enrichment of this pathway indicates that the efficiency of converting fatty acids to fatty acyl-CoA was enhanced in the muscle of the GS group, promoting the incorporation of fatty acids into triglycerides and membrane phospholipids and thereby supporting IMF deposition. Satoh et al. [42] found that pantothenate and CoA enhance fatty acid biosynthesis. In this study, the contents of various saturated and unsaturated fatty acids were significantly higher in the GS group, consistent with the enhanced CoA pathway.

In summary, feeding HMC shifted muscle energy metabolism toward enhanced oxidative capacity and lipogenesis by coordinately upregulating the TCA cycle, Valine, Leucine and Isoleucine Biosynthesis, taurine metabolism, and CoA biosynthesis, thereby promoting IMF deposition, improving tenderness, and enriching beneficial fatty acids. These mechanistic links provide a molecular-level explanation for the improved meat quality of Kazakh rams fed HMC.

### 4.5. Effects of Feeding HMC on Gut Microbiota Diversity in Kazakh Rams

Although the GS diet did not significantly alter alpha diversity of gut microbiota, Beta diversity showed a clear separation, indicating a shift in microbial community composition. Shang, S et al. [43] also found no difference in rumen microbial Alpha diversity in a study feeding HMC to dairy cows. At the phylum level, *Proteobacteria* is an important phylum in the gut microbiota. Ma, L et al. [44] also found in a study on microbial influences on fat deposition in pigs that intramuscular fat content was positively correlated with increased abundance of *Proteobacteria* in the pig gut microbiota; Guo, T et al. [45] also found in a study on yak meat quality during different grazing periods that meat quality was associated with a significant increase in the abundance of *Proteobacteria* and *Acidobacteriota* in the rumen; *Actinobacteria* plays a significant role in lignocellulose degradation and decomposition; Cao, Q et al. [46] showed that the relative abundance of *Acidobacteriota* is proportional to fatty acid synthesis. In summary, feeding HMC promotes fatty acid synthesis by altering the abundances of *Proteobacteria* and *Acidobacteriota* at the gut microbiota phylum level. At the genus level, *Ureaplasma* is one of the smallest pathogenic bacteria and can cause respiratory infections, indicating that lower abundance of this genus can improve host health [47]; the richness of the genus Comamonas has significantly increased at the genus level, making it a dominant genus in the gut microbiota; *Achromobacter* has strong antifungal activity and can improve gut health [48]. In summary, feeding HMC significantly alters the abundances of *Comamonas_F_589250* and *Achromobacter* at the gut microbiota genus level, thereby improving gut health, promoting the absorption of intestinal nutrients, and consequently enhancing fat synthesis.

### 4.6. Correlation Analysis Between Gut Microbiota at the Phylum Level and Intramuscular Fatty Acid Content in Kazakh Rams

Correlation analysis between phylum-level microbiota and fatty acid content found that *Firmicutes_A* was significantly negatively correlated with most fatty acids, such as Methyl docosatetraenoate, Methyl alpha linolenate, and Methyl vaccenate. Gulshara Abildinova et al. [49] found in their research that Firmicutes-A in the gut microbiota was negatively correlated with lipids such as triglycerides, which was consistent with the results of this experiment; *Proteobacteria* showed extremely significant positive correlations with fatty acids such as Methyl docosatetraenoate, Methyl vaccenate, and Methyl palmitoleate. Zhang, T et al. [50] confirmed that *Proteobacteria* mainly regulated fatty acid synthesis by modulating sugar metabolism; Wen, C et al. [51] also found that the relative abundance of *Proteobacteria* in the gut microbiota was positively correlated with fat deposition in yellow-feathered broilers. *Bacteroidota* showed significant positive correlations with fatty acids such as Methyl linoleate and Methyl arachidonate. Li, C et al. [52] indicated in a study on feeding silage to Hu sheep that *Bacteroidota* is related to the digestion and absorption of nutrients and fat deposition in sheep; *Chloroflexota* showed significant positive correlations with fatty acids such as Methyl myristoleate and Methyl palmitoleate. Bovio-Winkler, P et al. [53] found that the phylum *Chloroflexi* promotes the degradation of carbon compounds in anaerobic environments. In summary, the GS diet may promote the synthesis and deposition of body fatty acids by reducing the relative abundance of *Firmicutes-A* while increasing the abundances of microorganisms such as *Proteobacteria*, *Bacteroidota*, and *Chloroflexota*.

## 5. Conclusions

The results of this study indicate that replacing half of the ordinary crushed corn with HMC in the diet of Kazakh rams had no significant effect on overall slaughter performance except for backfat thickness, but significantly improved meat quality. Specifically, muscle shear force was significantly reduced, indicating improved tenderness, while intramuscular fat content was significantly increased. In addition, the levels of several beneficial fatty acids, such as oleic acid and α-linolenic acid, were significantly elevated. Muscle metabolomics analysis revealed that these changes in meat quality were associated with the enrichment of pathways related to valine, leucine and isoleucine biosynthesis, taurine and hypotaurine metabolism, pantothenate and CoA biosynthesis, and the tricarboxylic acid cycle. Gut microbiota analysis showed that HMC feeding was accompanied by a significant alteration in beta diversity, with *Firmicutes_A* being negatively correlated with most fatty acids and Proteobacteria being positively correlated with multiple fatty acids. In conclusion, partial replacement of ordinary corn with HMC is an effective strategy to improve the tenderness and nutritional quality of mutton in Kazakh rams, and this effect may involve coordinated changes in muscle energy metabolism and gut microbiota composition. Further interventional studies are needed to validate the causal relationships suggested by these associations.

## Figures and Tables

**Table 1:** Feed formulation and nutrient level of basic diet (DM %).

Items	Treatment ^(1)^	
	CT	GS
Roughage ingredients, %		
Whole plant corn silage	30.00	30.00
Cotton residue	30.00	30.00
Concentrate ingredients, %		
Ordinary crushed corn	24.00	12.00
High-moisture corn	0.00	12.00
Cottonseed meal	5.00	5.00
Soybean meal	5.00	5.00
Wheat bran	3.20	3.20
Premix ^(2)^	2.00	2.00
NaCl	0.40	0.40
NaHCO_3_	0.40	0.40
Total	100.00	100.00
Nutritional level ^(3)^		
GE (MJ/kg)	15.81	15.83
CP (%)	13.05	13.07
EE (%)	4.21	4.34
ADF (%)	25.35	26.19
NDF (%)	67.25	68.01
Ash (%)	8.35	8.64
Ca (%)	0.66	0.67
P (%)	0.34	0.35

^(1)^ The CT group was fed a basal diet containing 24% ordinary crushed corn (on a dry matter basis), while the GS group was fed a basal diet in which half of the ordinary crushed corn was replaced with HMC, resulting in a concentrate containing 12% ordinary crushed corn and 12% HMC. ^(2)^ The premix provided per kilogram of diet contained: vitamin A, 130,000–250,000 IU; vitamin D_3_, 40,000–100,000 IU; vitamin E, ≥1400 IU; copper (from tribasic copper chloride), 550–800 mg; iron (from ferrous sulfate), 1500–7000 mg; manganese (from manganese sulfate), 1100–3000 mg; zinc (from zinc sulfate), 800–2000 mg; iodine (from calcium iodate), 20–30 mg; selenium, 8–12 mg; cobalt, 20–30 mg; calcium, 10–20%; total phosphorus, 1.5%; sodium chloride, 12–20%; and moisture, ≤10.0%. All nutrient composition values are calculated values. ^(3)^ All nutrient levels are presented on a dry matter basis; nutritional levels are measured values [10]. GE, gross energy; CP, crude protein; EE, ether extract; ADF, acid detergent fiber; NDF, neutral detergent fiber; Ca, calcium; P, phosphorus.

**Table 2:** Effects of Feeding HMC on the Meat Production Performance of Kazakh Rams.

Items	Treatment ^(1)^		*p*-Value
	CT	GS	
Pre-slaughter live weight (kg)	59.23 ± 9.21	61.94 ± 9.99	0.636
Carcass weight (kg)	26.44 ± 3.57	28.56 ± 5.27	0.433
Net meat weight (kg)	22.23 ± 3.41	23.93 ± 4.21	0.461
Head and hoof weight (kg)	4.46 ± 0.98	4.93 ± 1.02	0.431
Tail fat weight (kg)	4.21 ± 0.72	4.63 ± 1.50	0.545
Dressing percentage (%)	44.63 ± 2.26	46.11 ± 1.89	0.347
Net meat rate (%)	37.53 ± 1.26	38.63 ± 1.15	0.167
Eye muscle area (cm^2^)	15.89 ± 0.97	16.48 ± 0.25	0.471
Back-fat thickness (cm)	0.77 ± 0.06 ^b^	0.92 ± 0.30 ^a^	0.021

^(1)^ The CT group was fed a basal diet containing 24% ordinary crushed corn (on a dry matter basis), while the GS group was fed a basal diet in which half of the ordinary crushed corn was replaced with HMC, resulting in a concentrate containing 12% ordinary crushed corn and 12% HMC; ^a,b^: Within the same row, numerical differences with different lowercase superscript letters indicate statistically significant differences (*p* < 0.05). Numerical values without superscript labels show no significant differences (*p* > 0.05).

**Table 3:** Effects of HMC on Physicochemical Properties of Mutton from Kazakh Rams.

Items	Treatment		*p*-Value
	CT	GS	
pH 24h ^(1)^	5.55 ± 0.04	5.65 ± 0.07	0.12
L*24h ^(2)^	34.01 ± 1.66	32.63 ± 0.76	0.27
a*24h ^(3)^	17.10 ± 2.26	15.80 ± 0.36	0.38
b*24h ^(4)^	5.77 ± 0.51	5.13 ± 0.06	0.11
Shear force (N/cm^2^)	40.34 ± 7.69 ^A^	17.95 ± 2.81 ^B^	<0.01
Drip loss (%)	0.52 ± 0.18	0.48 ± 0.17	0.77
Intramuscular protein content (%)	22.58 ± 0.94	21.67 ± 0.99	0.06
Intramuscular fat content (%)	6.23 ± 0.21 ^B^	9.74 ± 0.12 ^A^	<0.01

^(1)^ pH 24h: The pH value of the muscle measured at 24 h postmortem; ^(2)^ L*24h: Lightness of the muscle at 24 h postmortem; higher values indicate a paler (pale, soft, exudative) color, while lower values indicate a darker color; ^(3)^ a*24h: Redness of the muscle at 24 h postmortem; positive values indicate a redder color, associated with myoglobin content and oxygenation state; ^(4)^ b*24h: Yellowness of the muscle at 24 h postmortem; positive values indicate a more yellow color, often related to lipid oxidation or dietary components. ^A,B^: Numerical differences with different uppercase superscript letters indicate significantly higher values (*p* < 0.01). Numerical values without superscript labels show no significant differences (*p* > 0.05).

**Table 4:** Effects of Feeding HMC on the Amino Acid Content in the Muscle of Kazakh Rams.

Items	Treatment		*p*-Value
	CT (ng/mg)	GS (ng/mg)	
Alanine	963.62 ± 79.29	999.02 ± 64.41	0.42
Arginine	78.48 ± 15.15 ^a^	59.12 ± 7.98 ^b^	0.02
Asparagine anhydrous	27.25 ± 1.31	28.65 ± 3.07	0.32
Aspartic acid	114.80 ± 19.01 ^A^	85.92 ± 7.90 ^B^	<0.01
Glutamine	4803.03 ± 1033.84 ^a^	3596.33 ± 594.80 ^b^	0.03
Glutamic acid	13.59 ± 2.74	10.97 ± 2.13	0.09
Glycine	60.20 ± 8.37 ^b^	79.11 ± 13.14 ^a^	0.01
Histidine	120.13 ± 11.46	124.93 ± 9.49	0.44
Isoleucine	12.80 ± 1.27	13.10 ± 1.13	0.67
Cysteine	3.99 ± 1.01	4.33 ± 1.09	0.59
Leucine	34.94 ± 2.50	34.85 ± 4.19	0.96
Hydroxyproline	4.75 ± 0.39	5.72 ± 1.70	0.21
Tryptophan	7.42 ± 0.95	7.45 ± 0.53	0.94
Lysine	53.05 ± 5.58	49.48 ± 5.06	0.27
Methionine	6.38 ± 1.49	6.66 ± 0.68	0.68
Phenylalanine	15.61 ± 2.05	15.11 ± 0.93	0.59
Proline	18.44 ± 1.87	19.73 ± 1.35	0.21
Serine	19.99 ± 3.67	22.13 ± 5.31	0.44
Threonine	64.05 ± 3.19	65.51 ± 2.25	0.38
Tyrosine	13.61 ± 1.59	15.62 ± 1.61	0.05
Valine	16.51 ± 1.54	16.36 ± 1.35	0.86

^a,b^: Within the same row, numerical differences with different lowercase superscript letters indicate statistically significant differences (*p* < 0.05). ^A,B^: Numerical differences with different uppercase superscript letters indicate significantly higher values (*p* < 0.01). Numerical values without superscript labels show no significant differences (*p* > 0.05).

**Table 5:** Fatty Acid Content (μg/mg wet tissue) and Relative Percentage (%) of Total Fatty Acids in the Longissimus Muscle of Kazakh Rams.

Items	Treatment				*p*-Value
	CT (μg/mg)	CT (%)	GS (μg/mg)	GS (%)	
Methyl caproate	0.24 ± 0.026	0.01	0.24 ± 0.07	0.01	0.99
Methyl caprylate	0.68 ± 0.13	0.02	0.71 ± 0.34	0.01	0.81
Methyl caprate	4.79 ± 0.65 ^b^	0.12	7.90 ± 2.09 ^a^	0.11	0.02
Methyl undecanoate	0.22 ± 0.01 ^B^	0.01	0.29 ± 0.03 ^A^	0.01	<0.01
Methyl laurate	2.29 ± 0.89 ^b^	0.06	6.98 ± 1.65 ^a^	0.1	0.04
Methyl tridecanoate	0.40 ± 0.04 ^b^	0.01	0.62 ± 0.09 ^a^	0.01	0.04
Methyl myristate	51.85 ± 8.06 ^B^	1.28	130.28 ± 18.77 ^A^	1.84	<0.01
Methyl myristoleate	1.49 ± 0.20	0.04	1.83 ± 0.32	0.03	0.25
Methyl pentadecanoate	9.54 ± 0.98 ^b^	0.24	19.96 ± 2.49 ^a^	0.28	0.04
Methyl palmitate	884.90 ± 189.32 ^B^	21.92	1621.25 ± 354.04 ^A^	23.03	<0.01
Methyl Palmitelaidate	2.67 ± 0.18	0.07	2.85 ± 1.09	0.04	0.69
Methyl palmitoleate	37.49 ± 7.02 ^b^	0.93	78.47 ± 9.50 ^a^	1.11	0.04
Methyl heptadecanoate	34.24 ± 6.15 ^B^	0.85	68.28 ± 15.81 ^A^	0.97	<0.01
Methyl stearate	818.24 ± 22.87 ^b^	20.27	1325.83 ± 71.55 ^a^	18.83	0.01
Methyl elaidate	19.51 ± 1.19	0.48	20.23 ± 2.44	0.29	0.76
Methyl transvaccenate	43.36 ± 3.14 ^b^	1.07	92.16 ± 9.57 ^a^	1.31	0.04
Methyl oleate	1364.07 ± 104.43 ^b^	33.8	2757.68 ± 278.16 ^a^	39.16	0.04
Methyl vaccenate	112.25 ± 28.38 ^b^	2.78	222.19 ± 46.12 ^a^	3.16	0.03
Methyl 10-transnonadecenoate	15.46 ± 1.27	0.38	18.17 ± 2.96	0.26	0.32
Methyl linoleate	428.15 ± 43.43	10.61	433.74 ± 39.70	6.16	0.82
Methyl arachidate	4.84 ± 0.28	0.12	5.64 ± 0.51	0.08	0.15
Methyl gamma linolenate	6.43 ± 0.30	0.16	6.54 ± 0.53	0.09	0.65
Methyl alpha-linolenate	35.13 ± 5.39 ^B^	0.87	52.48 ± 8.51 ^A^	0.75	<0.01
Methyl heneicosanoate	3.60 ± 0.16	0.09	4.03 ± 0.66	0.06	0.21
Methyl 11-14 eicosadienoate	6.10 ± 0.24	0.15	6.40 ± 0.49	0.09	0.21
Methyl behenate	1.49 ± 0.09	0.04	1.46 ± 0.05	0.02	0.58
Methyl 11-14-17 eicosatrienoate	3.35 ± 0.28	0.08	3.32 ± 0.18	0.05	0.86
Methyl arachidonate	85.18 ± 6.58	2.11	88.75 ± 6.17	1.26	0.38
Methyl eicosapentaenoate	19.50 ± 0.54	0.48	19.66 ± 1.08	0.28	0.76
Methyl docosatetraenoate	17.28 ± 0.37 ^b^	0.43	18.13 ± 0.65 ^a^	0.26	0.02
All-cis-4,7,10,13,16-docosapentaenoic acid	20.96 ± 1.64 ^B^	0.52	23.84 ± 0.40 ^A^	0.34	<0.01
Total Fatty Acid	4035.70 ± 56.81	100	7039.91 ± 107.16	100	0.78

^a,b^: Within the same row, numerical differences with different lowercase superscript letters indicate statistically significant differences (*p* < 0.05). ^A,B^: Numerical differences with different uppercase superscript letters indicate significantly higher values (*p* < 0.01). Numerical values without superscript labels show no significant differences (*p* > 0.05).

**Table 6:** Effects of Feeding HMC on Gut Microbiota Alpha Diversity in Kazakh Rams.

Items	Treatment		*p*-Value
	CT	GS	
ACE ^(1)^	468.43 ± 79.33	414.67 ± 116.17	0.33
Chao1^(2)^	469.33 ± 80.59	415.86 ± 117.53	0.34
Shannon ^(3)^	6.72 ± 0.62	6.19 ± 0.82	0.19
Simpson ^(4)^	0.96 ± 0.03	0.93 ± 0.05	0.29

^(1)^ ACE (Abundance-based Coverage Estimator) is an index used to estimate the number of species in a community. ^(**2**)^ Chao1 index: Estimates the total number of species in a community, primarily reflecting species richness. A higher value indicates greater species richness and a larger potential number of undiscovered species. ^(3)^ Shannon index: Incorporates both species richness and the evenness of species abundance. A higher value indicates greater species richness, more uniform distribution of individuals, higher uncertainty, and thus greater overall diversity. ^(4)^ Simpson index: comprehensively reflects both species richness and evenness. A value closer to 1 suggests not only higher species richness but also a more uniform distribution of individuals among species, with no single dominant species.

## Data Availability

The data presented in this study are available on request from the corresponding author.

## Abbreviations

The following abbreviations are used in this manuscript:

HMC	High Moisture Corn
CT	Control Group (fed a basal diet containing 24% ordinary crushed corn on a dry matter basis)
GS	Experimental Group (fed a basal diet in which half of the ordinary crushed corn was replaced with HMC, resulting in a concentrate containing 12% ordinary crushed corn and 12% HMC)
GE	Gross Energy
Ca	Calcium
P	Phosphorus
EE	Ether Extract
ADF	Acid Detergent Fiber
NDF	Neutral Detergent Fiber
DM	Dry Matter
KEGG	Kyoto Encyclopedia of Genes and Genomes
H&E	Hematoxylin and Eosin
IMFAs	Intramuscular Fatty Acids
AOAC	Association of Official Agricultural Chemists
BCAAs	Branched-Chain Amino Acids
PCA	Principal Component Analysis
OPLS-DA	Orthogonal Partial Least Squares-Discriminant Analysis
OTU	Operational Taxonomic Unit
LEfSe	Linear Discriminant Analysis Effect Size
NMDS	Non-metric Multidimensional Scaling
VIP	Variable Importance in Projection
NH_3_-N	Ammonia Nitrogen
UDP	Uridine Diphosphate
